# Autocorrection – how to measure the skills acquired during physical therapy sessions

**DOI:** 10.1186/1748-7161-7-S1-P20

**Published:** 2012-01-27

**Authors:** HR Weiss, S Seibel

**Affiliations:** 1 Orthopedic Rehabilitation Services, Gensingen, Germany

## Background

It is common sense that physiotherapy in the treatment of scoliosis patients should improve the skills for active self-correction of the individual patient [1,2]. Although the autocorrection (AC) patients can achieve when they use certain high correction exercises obviously can be observed, there is no tool to enable the measuremet of this patient skill. Aim of this study was to test whether AC can be measured with the help of the Scoliometer (ATR) [3].

## Materials and methods

9 Patients with Idiopathic Scoliosis (2 males and 7 females) (IS) with an average Cobb angle of 46° (29 – 64°) and with an average age of 14 (11 – 18) years underwent a five days course of Scoliosis Short-Term Rehabilitation (SSTR). ATR (Angle of Trunk Rotation = Scoliometer) measurements were taken before and after the treatment. Additionally, the ability to correct themselves (AC) was measured after four days of treatment.

## Results

The ATR was reduced significantly from 10.3° to 8.2° (p < 0,001) after treatment in the nine patients with scoliosis. The ability to correct themselves (AC) as measured with the help of the Scoliometer (ATR 8.2° / ATR 5.7° auto-corrected without additional help by the therapist) was 1,45 and the difference between ATR 8.2° / ATR auto-corrected 5.7° was significant as well (p = 0,0035).

## Conclusions

Measurement of autocorrection is possible. The relation ATR / ATR autocorr. will usually be 1 (no autocorrection possible) at the start of the very first specific treatment and may increase when the patient gains the necessary exercising skills.
